# Observations on the use of Bruton’s tyrosine kinase inhibitors in SAR-CoV-2 and cancer

**DOI:** 10.1186/s13045-020-00999-8

**Published:** 2021-01-13

**Authors:** Brooke Benner, William E. Carson

**Affiliations:** 1 Comprehensive Cancer Center, The Ohio State University, Columbus, OH USA; 2 Department of Surgery, Division of Surgical Oncology, The Ohio State University, N924 Doan Hall, 410 W. 10th Ave, Columbus, OH 43210 USA

**Keywords:** Bruton’s tyrosine kinase, Sars-CoV-2, COVID-19, Cancer, Ibrutinib, Acalabrutinib, IL-1β

## Abstract

Bruton’s tyrosine kinase (BTK) inhibitors, drugs utilized in cancer, are being repurposed for severe acute respiratory syndrome coronavirus-2 (SARS-CoV-2) (COVID-19). Recently, BTK inhibitors acalabrutinib and ibrutinib have been found to protect against pulmonary injury in a small group of patients infected with SARS-CoV-2. The high levels of pro-inflammatory cytokines found in the circulation of COVID-19 patients with severe lung disease suggest the involvement of the innate immune system in this process. Understanding the potential mechanism of action of BTK inhibition in SARS-CoV-2 is clearly of importance to determine how acalabrutinib, ibrutinib and possibly other BTK inhibitors may provide protection against lung injury.

To the Editor:

SARS-CoV-2 infects nasal and respiratory epithelial cells via attachment to angiotensin-converting enzyme 2 (ACE2) [1, 2] which in turn produces factors conducive to the recruitment of monocytes into the pulmonary system [3]. In a subset of patients, an inflammatory macrophage response is activated which leads to respiratory failure. Indeed, the postmortem examination of the lungs in patients that succumb to SARS-CoV-2 reveals the presence of an extensive cellular infiltration dominated by macrophages [3]. The subsequent infection of recruited pulmonary macrophages could be enhanced via increased surface expression of ACE2 induced by locally produced interferon alpha. Once infected, macrophages can sense the single-stranded RNA of the SARS-CoV-2 virus via Toll-like receptor 7 with downstream signaling mediated by nuclear factor kappa beta (NF-κβ) which drives the transcription of pro-inflammatory genes such as IL-6 [4]. In addition, the presence of the virus will be sensed by the NLRP3 inflammasome, which activates caspases that cleave pro-IL-1β and permit the release of the mature protein into the surrounding environment [5, 6]. Previously, our group demonstrated that myeloid-derived suppressor cells (MDSC) express BTK and that their generation, migration and antitumor activity could be inhibited with ibrutinib [7]. More recently, Benner et al. from our group have shown that tumor-associated macrophages (TAM) express BTK and that this enzyme appears to physically interact with the NLRP3 inflammasome upon activation of TAM with LPS and ATP. It was shown that inhibition of BTK with ibrutinib could inhibit inflammasome activation in TAMs and reduce the release of mature IL-1β [8]. Notably, Ito *et al.* have also demonstrated that BTK is essential for NLRP3 inflammasome activation and contributes to ischemic brain injury [9]. These findings suggest a potential mechanism by which ibrutinib can inhibit macrophage activation, lower the production of IL-1β and alter the pulmonary inflammatory landscape in patients infected with SARS- CoV-2. Inhibition of the inflammasome with ibrutinib did not reduce the release of IL-6 in our model system, suggesting that the effects of BTK inhibition are targeted in nature. Indeed, high systemic levels of IL-6 are often found in patients with severe SARS-CoV-2 infection, while increased levels of circulating IL-1β either were not detected or were found to be elevated at low levels (1–8 pg/mL) in ICU and non-ICU patients, as compared to normal individuals. These reports indicate that the effects of IL-1β might be more local in nature and active via autocrine and/or paracrine pathways [10–12]. Indeed, the immunohistochemical examination of lung tissues from patients that succumbed to COVID-19 revealed that lung epithelial cells and monocytes/macrophages expressing both ACE2 and the SARS‐CoV S protein reacted strongly with monoclonal antibodies to IL‐1β, IL‐6 and TNF‐α [13]. Previously, it has been shown in murine models of influenza viral infection, pneumococcal pneumonia and cecal ligation and puncture (CLP)-induced sepsis that BTK inhibition with ibrutinib prevented lung injury and led to reduced alveolar macrophage activation, neutrophil influx and cytokine release [14–16].

A clinical study was recently conducted that reported on six Waldenstrom macroglobulinemia patients who were taking the BTK inhibitor ibrutinib and were subsequently diagnosed with COVID- 19. These six patients exhibited only mild COVID-19-related symptoms [17]. In this study, five patients received 420 mg/d of ibrutinib and one patient received a reduced dose of 140 mg/d. Notably, the patients receiving the 420 mg/d did not require hospitalization and experienced steady improvement and resolution or near resolution of COVID-19–related symptoms during the follow-up [17]. A second clinical study administered acalabrutinib off-label to 19 patients hospitalized with severe COVID-19 (11 on supplemental oxygen; eight on mechanical ventilation) [12]. Following 10–14 days of treatment, acalabrutinib was found to improve oxygenation, demonstrated by the ability of eight of 11 patients in the supplemental oxygen cohort being discharged on room air, and four of eight patients in the mechanical ventilation cohort undergoing successfully extubation [12]. Notably, IL-6 production by monocytes was increased in patients with severe COVID-19 compared to healthy volunteers. Taken together, these findings support the hypothesis that the release of pro-inflammatory cytokines by pulmonary macrophages in SARS-CoV-2 patients is a major contributor to pulmonary disease and that BTK inhibition with ibrutinib or acalabrutinib could provide some degree of protection against lung injury in this setting. Still, given the multiple factors that are produced during the course of severe SARS-CoV-2 infection, the blockade of multiple inflammatory pathways might be required to ameliorate the clinical picture of patients with severe pulmonary disease due to SARS-CoV-2. Similarly, the reversal of the pro-tumor actions of MDSC and TAM within the tumor microenvironment may require multiple interventions that target pro-inflammatory pathways. The utility of BTK inhibitors in treating COVID-19 is being investigated in several off-label clinical studies. These studies include the use of acalabrutinib co-administered with a proton pump inhibitor (NCT04497948), acalabrutinib together with best supportive care (NCT04380688, NCT04346199), ibrutinib in patients requiring hospitalization (NCT04439006) and a clinical study to evaluate the pathogenesis of BTK-mediated hyper-inflammatory responses (NCT04394884) in patients with COVID-19. It should be noted that definitive information is pending, and the off-label use of drugs for the treatment for COVID-19 is not recommended at this time.

## Data Availability

Not applicable.
